# Biogeography and Phylogeny of Wood-feeding Cockroaches in the Genus *Cryptocercus*

**DOI:** 10.3390/insects2030354

**Published:** 2011-07-19

**Authors:** Kiyoto Maekawa, Christine A. Nalepa

**Affiliations:** 1 Graduate School of Science and Engineering, University of Toyama, 3190 Gofuku, Toyama 930-8555, Japan; 2 Department of Entomology, North Carolina State University, Raleigh, NC 27695-7613, USA; E-Mail: christine_nalepa@ncsu.edu

**Keywords:** woodroaches, molecular phylogeny, molecular clock, chromosome number

## Abstract

Subsocial, xylophagous cockroaches of the genus *Cryptocercus* exhibit a disjunct distribution, with representatives in mature montane forests of North America, China, Korea and the Russian Far East. All described species are wingless and dependent on rotting wood for food and shelter at all stages of their life cycle; consequently, their distribution is tied to that of forests and strongly influenced by palaeogeographical events. Asian and American lineages form distinct monophyletic groups, comprised of populations with complex geographic substructuring. We review the phylogeny and distribution of *Cryptocercus*, and discuss splitting events inferred from molecular data.

## 1. Introduction

**Figure 1:**
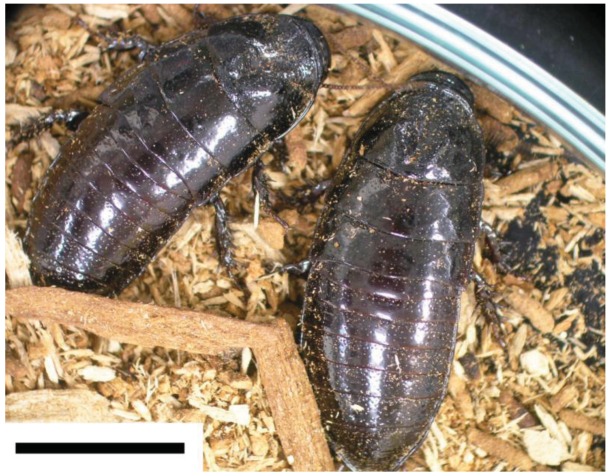
Pairs of *Cryptocercus punctulatus* collected at Mt. Collins (male chromosome number 2n = 45), North Carolina, eastern United States. Scale bars indicate 10 mm.

Woodroaches of the genus *Cryptocercus* (*Dictyoptera: Cryptocercidae*) are subsocial, xylophagous cockroaches that occur in mountain forests of temperate regions, living in galleries that they chew within rotten logs [1–5]. Numerous recent phylogenetic studies of both the insects and their symbionts strongly suggest that the genus is a sister group to termites (reviewed in [6]). The genus has a disjunct distribution, occurring in eastern and western North America, and in China, Korea and the Russian Far East. All *Cryptocercus* spp. are wingless cockroaches (see Figure 1), and depend on dead wood for food and shelter at all stages of their life cycle. It is therefore likely that their distributional pattern has been strongly affected by palaeogeographic events that influenced their source tree hosts, such as the appearance of land bridges or the uplift of mountains. Both North American [7–11] and Asian taxa [10,12] have been the subjects of independent phylogeographic analyses, and the phylogenetic relationships and divergence times within and between Palaearctic and Nearctic taxa have been analyzed based on molecular data [13]. Here we review the distribution and phylogeny of *Cryptocercus* (1) worldwide, (2) in the Palaearctic, and (3) in the Nearctic.

## 2. Worldwide Distribution and Phylogeny of *Cryptocercus*

### 2.1. When did the Splitting Events Occur?

**Figure 2:**
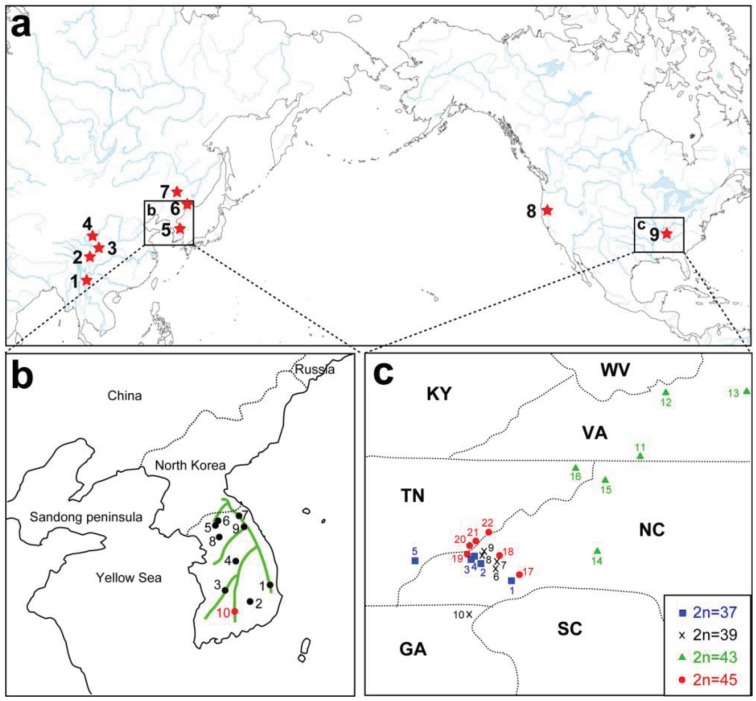
(**a**) Geographic distribution of *Cryptocercus* spp. in the world (type locality is shown). 1: *C. meridianus* in China (Yunnan) [15]. 2: *C. matilei* in China (Sichuan) [16]. 3: *C. primarius* in China (Sichuan and Yunnan) [17,18]. 4: *C. hirtus* in China (Gansu) [15]. 5: *C. kyebangensis* in South Korea [4]. 6: *C. relictus* in Russia (Ussuri region) [19]. 7: *C. parvus* in China (Mudanjiang) [15]. 8: *C. clevelandi* in North America (Oregon) [7]. 9: *C. punctulatus* in the Appalachian Mountains [1,7]. Burnside *et al.* [8] named 3 new species (*C. wrighti*, *C. darwini* and *C. garcial*) in this eastern North American group, but the validity of these has been questioned ([20]; see the section 3.1); (**b**) Solid black and red circles indicate the localities of South Korean *Cryptocercus* used in Maekawa *et al.* [13]. Location numbers correspond to sites shown in Figure 3. Green lines represent mountains; (**c**) Distribution of male karyotypes of the *C. punctulatus* species complex in the Southern Appalachian Mountains, with state boundaries indicated (22 sites used in Everaerts *et al.* [11]). Location numbers correspond to sites shown in Figure 4.

A remarkable feature of the present geographic distribution of *Cryptocercus* is the disjunct distribution found between the Nearctic and Palearctic regions, and between eastern and western North America (Figure 2A). Grandcolas [14] hypothesized that the American lineage evolved from Asian taxa. If this hypothesis is supported, then Asian taxa would be ancestral to the American lineage, with the latter as apical. However, independent analyses using multiple representatives of both North American and Asian taxa show that the two groups form respective monophyletic groups (e.g., [12]).

There are several hypotheses regarding the divergence times between Asian and American taxa. Grandcolas [14] proposed that “the ancestor of *Cryptocercus* was distributed in Asia, extending posteriorly its distribution to North America”. He hypothesized that these movements occurred via the connection of the two landmasses during the late Tertiary. Later, Grandcolas *et al.* [4] proposed that the splitting events between Asian and American species occurred between ∼18 and 2 million years ago (MYA), based on the genetic divergence of partial mitochondrial rDNA sequences.

Clark *et al.* [9] proposed a markedly different scenario: “During the Jurassic (213-144 MYA), the ancestor of extant *Cryptocercus* inhabited the temperate deciduous forests of the Arcto-Tertiary complex in the extreme northern regions of the Northern Hemisphere…A general cooling trend began during the Cretaceous (144–65 MYA), which forced the Arcto-Tertiary community (and the ancestor of *Cryptocercus*) to move south into Asia and North America”. In formulating their hypothesis, Clark *et al.* [9] utilized data from not only mitochondrial and nuclear genes of the cockroaches, but also from 16S rRNA genes of their fat body endosymbionts (*Blattabacterium* spp.). These endosymbionts have been found in all cockroaches examined to date (with one exception-Nocticolidae), and just one termite species, the basal taxon *Mastotermes darwiniensis* (reviewed in [6]). Clark *et al.* [9] demonstrated topological congruence between the endosymbionts and their hosts, and using a 0.6–1.0% per 50 million years sequence divergence rate, estimated the divergence times between Asian and American species at about 115–70 MYA. This sequence divergence rate was originally proposed by Bandi *et al.* [21], based on the assumption that cockroaches and termites are each monophyletic and that they diverged from each other sometime between 250 and 135 MYA. However, Maekawa *et al.* [13] pointed out that the former hypothesis was not supported by more recent phylogenetic studies among cockroaches, termites and mantids (which together form the Dictyoptera). Both molecular and morphological evidence show a sister-group relationship between termites and *Cryptocercus*, and the paraphyly of cockroaches in relation to termites (reviewed in [6]). Moreover, Maekawa *et al.* [13] pointed out that the fossil record was not consistent with the idea that termites and cockroaches diverged from each other 250 MYA. Cockroaches are one of the most ancient insect groups, and their fossils are observed in Carboniferous strata. However, cockroach fossils from the Carboniferous to the Jurassic possess a distinct, external ovipositor, while the ovipositors of more recent fossils and of extant Blattaria either do not exceed the tip of the abdomen (cockroaches) or are vestigial (termites except for *Mastotermes*). Consequently, ancient fossil cockroaches with external ovipositors are likely to be paraphyletic with respect to the Dictyoptera [22]. Indeed, termite, mantid and modern cockroach fossils first appear in early Cretaceous strata (∼130 MYA). Consequently, Maekawa *et al.* [13] suggested that a more realistic estimate for the split of Dictyoptera was sometime between 135 and 180 MYA. The older value was based on fossil records of the Mesoblattinidae, which has been proposed as the stem group of Dictyoptera [23,24].

The monophyly of Asian taxa could not be verified because in the studies of both Grandcolas *et al.* [4] and Clark *et al.* [9] only one Asian representative was used. Park *et al.* [12] estimated divergence times between Asian and American lineages based on the mitochondrial COII gene sequences of multiple Asian and two American taxa. They suggested that Asian and American lineages diverged around 61–26 MYA. These values are intermediate between those of Clark *et al.* [9] and Grandcolas *et al.* [4].

### 2.2. Endosymbiont 16S Phylogeny Reveals a Late Cretaceous-Early Tertiary Split of Asian and American Groups

**Figure 3:**
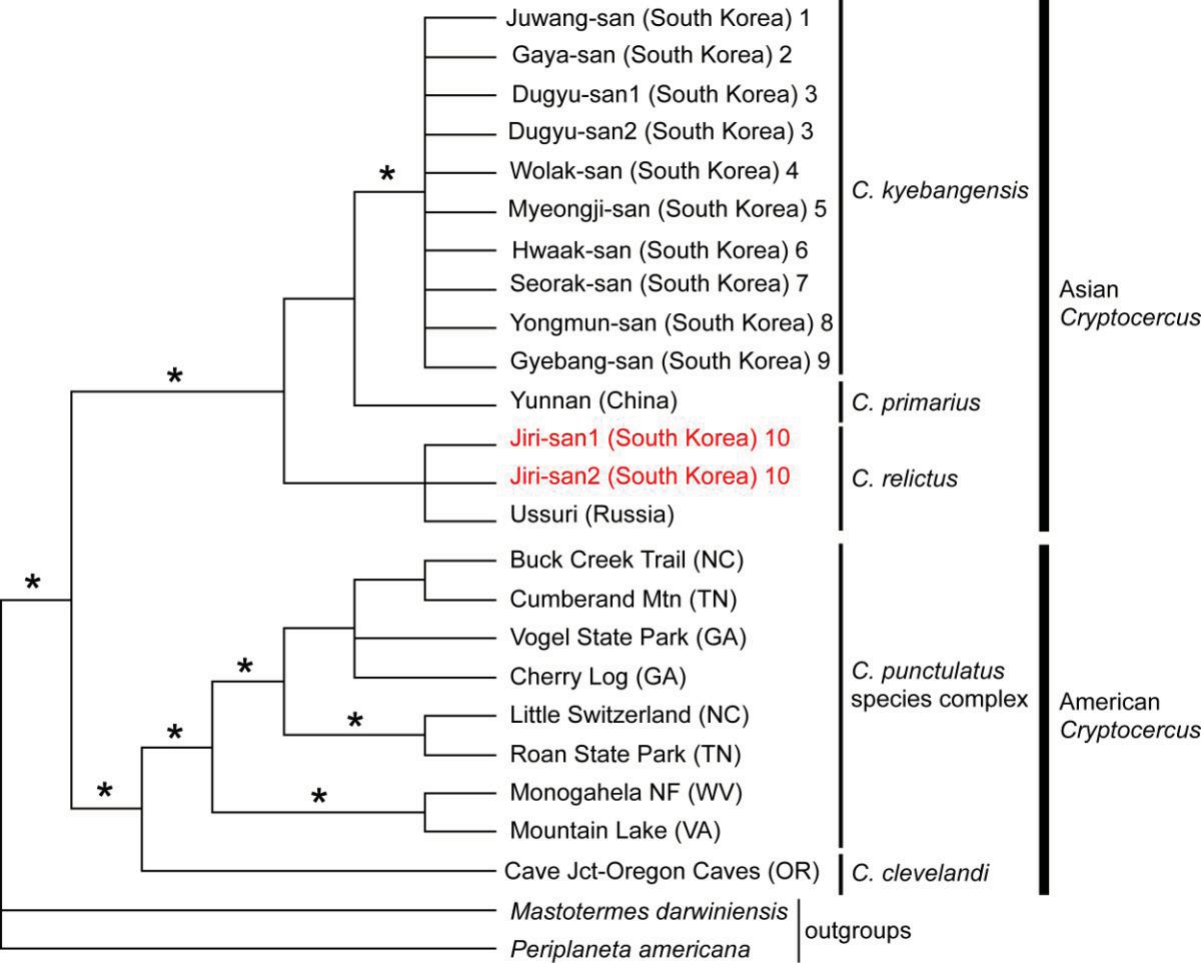
Phylogenetic relationships among endosymbionts of *Cryptocercus* spp. inferred from 16S rRNA gene sequences (re-drawn from the figure shown in Maekawa *et al.* [13]). Nodes with strong support (more than 90% Bayesian posterior probabilities and MP bootstrap probabilities) are indicated by an asterisk. Each terminal is labeled with the collection location name and species name. Collection location numbers are also indicated for the South Korean samples (shown in 2b).

No reports of molecular phylogenetic analyses using *Blattabacterium* endosymbionts from multiple individuals of Asian and American *Cryptocercus* existed until Maekawa *et al.* [13] estimated their phylogenetic relationships and divergence times using endosymbiont 16S sequences of Korean hosts combined with published data. The monophyly of Asian taxa, as well as that of American taxa, was supported by more than 90% Bayesian posterior probabilities and most parsimonious (MP) bootstrap probabilities (Figure 3). The data suggested, then, that there had been only one divergence event between Asian and American *Cryptocercus*.

Maekawa *et al.* [13] calculated the average maximum likelihood distance between endosymbiont 16S from *M. darwiniensis* and the *Cryptocercus* spp. (6.05 ± 0.57%, average ± SD, n = 15), and suggested that 0.0302 substitutions per site occurred in each of these lineages. Using an estimate for the split of the Dictyoptera (135 and 180 MYA; Section 2.1), they calculated that the evolutionary rate was 0.0084–0.0111 per site per 50 MY. Moran *et al.* [25] reported that the evolutionary rates of aphid endosymbiont 16S were 0.0076–0.0232 per site per 50 MY, and the above estimates of *Blattabacterium* 16S were within this range. Using this evolutionary rate, Maekawa *et al.* [13] suggested that the divergence times between Asian and American *Cryptocercus* (which have a mean ML distance of 2.61 ± 0.25% (n = 54)) were between 77.8–58.7 MYA.

These authors then addressed the correspondence between the estimated divergence times and hypotheses regarding the paleogeography of the region. During the late Cretaceous/early Tertiary (∼65 MYA), Laurasia had been subdivided into the Aquillapollenites Province (eastern Asia and Western North America) and the Normapolles Province (eastern North America and Europe) [26–28], and the temperate climates of the early Tertiary allowed a “boreotropical flora” to dominate the Northern Hemisphere [29]. The boreotropical flora spread between Eurasia and America during the early Eocene (∼55 MYA), aided by the existence of the North Atlantic land bridge and the Bering Land Bridge [28]. Although the source origin of the genus is still unclear, the results of both Maekawa *et al.* [13] and Park *et al.* [12] suggest that the ancestor of *Cryptocercus* had evolved by this point and existed in both Asia and America. Disruptions of both the Bering Land Bridge and the North Atlantic Bridge during the middle Eocene (∼50–45 MYA) (see [28]) probably contributed to the divergence between Asian and American *Cryptocercus*. The Bering Land Bridge may have reformed in the late Eocene [28], with Asia and America continuously connected with each other until the formation of the Bering Strait in the late Miocene [29–30]. The results suggest that gene flow between Asian and American *Cryptocercus* had been disrupted by the late Eocene, despite the continuous connection and warm climates of the two continents until the late Tertiary. Widespread grassland in these regions [29] might have contributed to this cessation of gene flow between Asian and American *Cryptocercus*.

## 3. Distribution and Phylogeny of Palaearctic *Cryptocercus*

### 3.1. Taxonomy and Phylogenetic Relationships

Currently, seven Asian species of *Cryptocercus* are recognized. *Cryptocercus primarius* and *C. matilei* are found in Sichuan Province, West China [16,17] and *C. primarius* was rediscovered in two forests within the Hengduan Mountains of the northwestern Yunnan Province, West China [18]. The other Asian species, *C. relictus*, is found in the Ussuri region and Siberia (Russia), eastern Manchuria, and South Korea [19,31]. A South Korean species, *C. kyebangensis*, was described by Grandcolas *et al.* [4]. Later, Grandocolas *et al.* [15] described three additional species, *C. hirtus*, *C. meridianus* and *C. parvus*, from China (see Figure 2A and 2B for the map).

The phylogeography of Northeast Asian *Cryptocercus* spp. was inferred from mitochondrial COII and 16S genes and nuclear 18S gene sequences [12]. The data show two distantly related groups in the Korean taxa: the southwestern population (*Cryptocercus* from Jiri-san) and the population containing the remaining Korean individuals (*C. kyebangensis*). The former was shown to be most closely related to the populations in Northeast China and eastern Russia (*C. relictus*). This is an unexpected finding, because Jiri-san is located in the extreme southwest region of the Korean Peninsula.

Lo *et al.* [10] subsequently sequenced mitochondrial COII and 16S genes in *C. primarius* to determine its phylogenetic position. Despite being geographically proximate to one another, *C. primarius* samples from the Yunnan region in China were genetically distant, and precise phylogenetic relationships among the Asian species (*C. primarius*, *C. relictus* and *C. kyebangensis* used in this study) were not clearly delineated.

### 3.2. Biogeography of Cryptocercus in South Korea

Park *et al.* [12] utilized COII transversion rates of 0.13–0.30%/MY [32] to infer the biogeography of Korean *Cryptocercus*. They suggested that the divergence of the three groups (*Cryptocercus* in Northeast China and eastern Russia, those in Jiri-san, and those in the remainder South Korea) occurred during the Miocene (7.5–17.4 MYA), and that the first and second populations diverged from each other sometime between 0.8–1.9 MYA. Based on present distributions, Park *et al.* [12] proposed that the common ancestor of extant *C. relictus* and *C. kyebangensis* may have been distributed in East China or Northeast China, and then migrated into the Korean Peninsula during the Miocene (7.5–17.4 MYA).

These authors pointed out two possible routes for the migration into the Korean Peninsula: via mountain chains that connect Manchuria to South Korea, or via the Yellow Sea basin. Regarding the first possible route, potential connections include the Taebaek Mountains in South Korea, and the Jangbai Mountains and Nangnim Mountains in North Korea. As to the second route, Park *et al.* [12] noted that *Cryptocercus* is found in some mountainous areas (e.g., Yongmun-san) in the western part of the Korean Peninsula, and that the western coastline of the Korean Peninsula is located only about 250 km from that of the Sandong Peninsula of East China. Furthermore, the Yellow Sea basin was above sea-level during the early-mid Miocene (especially, 11.5–16.5 MYA) [33]. If ancestral populations of *C. kyebnagensis* had migrated into Korea via the Yellow Sea basin, they could have later spread along the Taebaek Mountains during the Pleistocene (0.4–0.8 MYA).

Park *et al.* [12] offered two hypotheses for the unexpected distribution of *C. relictus* in Jiri-san. The first is that ancestral populations in Manchuria might have moved south into the Korean Peninsula. The divergence time (about 13.5 MYA) between populations in Manchuria and Jiri-san corresponds to the early Pleistocene, when advances and retreats of glaciers might have affected divergence between the populations. An alternate hypothesis is that, during the estimated time period (0.8–1.9 MYA), a portion of the ancestral population of extant *C. relictus* moved to Manchuria, whereas another population moved into the southwestern parts of South Korea via the Yellow Sea basin. The Yellow Sea basin was completely or partially exposed during the Pleistocene [34–36], and the bones of extinct animals (*Elephas namadicus* and *Mammuthus primigenius*) have been collected there (reviewed in [34]). It is therefore possible that ancestral populations of *C. relictus* (Jiri-san) in East China could have migrated into the southwestern part of the Korean Peninsula via a forested land route on the Yellow Sea basin.

## 4. Distribution and Phylogeny of Nearctic *Cryptocercus*

### 4.1. Taxonomy of Nearctic Species

Until 1997, *C. punctulatus* was the sole species reported in the United States. Its distribution, however, was strongly disjunct; an eastern population lived in the Appalachian Mountains from Pennsylvania to Alabama, and a western population inhabited the Pacific Northwest (Washington, Oregon, and northern California) [1,37] (see Figure 2A for the map). The distance separating the two populations (>3400 km), as well as the winglessness and consequent low vagility of the insect hinted at taxonomic division of the two populations. Cleveland *et al.* [1] noted that the western population of *C. punctulatus* had a longer developmental time, a larger body size, and 5 species of cellulolytic protozoa in the hindgut not found in the gut fauna of the Appalachian group. Later investigations documented that chromosome numbers (see the following section), mitochondrial gene sequences encoding 12S and 16S [38], and the chemistry of tergal gland secretions [39] also differed. Preliminary field studies suggested that the two populations may be reproductively isolated [38], and Nalepa *et al.* [7] reported not only differences in sequences of endosymbiont 16S, but also several biological differences between the two groups (egg number/ootheca, density of sternal punctation, male abdomen). These authors concluded that *C. punctulatus* as then represented in the literature included at least two species. The name *C. punctulatus* applied to populations in the eastern United States, and those in the northwestern United States were described as a new species, *C. clevelandi*.

Burnside *et al.* [8] later named three new species in the eastern population (*C. wrighti*, *C. darwini*, and *C. garciai*) based on differences in the sequences of mitochondrial genes (12S and 16S) and chromosome number. The validity of the species-level status proposed by these authors was questioned [20], however, because: (1) chromosome numbers were known for only part of the sample; (2) the evolutionary relationships among members of the species complex were unclear, (3) reproductive compatibility had not been investigated; and (4) although morphological variation was apparently present, it had not been demonstrated that this variation consistently distinguished the proposed species. Consequently, we do not use the species-level status proposed by Burnside *et al.* [8] for the populations in the eastern United States, but do recognize that *C. punctulatus* is a cryptic species complex.

### 4.2. Chromosome Evolution in the C. punctulatus Species Complex

Although karyotype studies consistently show that *C. clevelandi* has a diploid chromosome number of 48 in females (2n = 47 in males), four karyotypes have been detected in the *C. punctulatus* species complex. A diploid chromosome number of 40 for females (2n = 39 in males) was reported by Cohen and Roth [40] from the area of Highlands, North Carolina. Luykx [41] found males with 2n = 37 chromosomes, also from the Highlands area, and with 2n = 43 chromosomes at Mountain Lake, Virginia. A fourth chromosome number, 2n = 45, was reported from the Asheville, North Carolina, area [38], but the mid-point of the karyotype series (2n = 41) has not yet been detected. Palearctic species of *Cryptocercus* display a similar pattern of chromosomal variation in geographically proximate regions, with male chromosome numbers of both 2n = 19 and 2n = 21 reported from *C. primarius* samples collected in Yunnan Province, China [10]. Robertsonian changes that involve the fission or fusion of nonhomologous autosomes is responsible for the reported variations in chromosome number [41], and although these changes shift the number of chromosomes, they do not alter the fundamental genic content of the arms of the chromosomes involved. Either a chromosome with a median centromere is split into two chromosomes with terminal centromeres, or two chromosomes with terminal centromeres are fused into one chromosome with a centromere near the middle [42]. Because chromosome numbers of Palearctic *Cryptocercus* were approximately half those reported in Nearctic species, Lo *et al.* [10] suggested the possibility of either genome dupulication in the ancestor of the Nearctic lineage, or a reduction in the ancestor of the Palearctic lineage. There are reports of highly variable chromosome numbers among species of the other cockroach genera (*i.e.*, male 2n = 23–49 in *Blattella* spp., 34–50 in *Ischnoptera* spp., 38–74 in *Blaberus* spp., and 36–50 in *Epilampra* spp.) [40].

The geographic distributional pattern of the four known karyotypes (male 2n = 37, 39, 43, 45) of the *C. punctulatus* species complex was reported by Nalepa *et al.* [20], based on 71 sites in the Southern Appalachian Mountains with a concentration on western North Carolina. Cockroach populations with different karyotypes were geographically structured in a mosaic marked by abrupt geographic transitions between them, and with at least one karyotype (2n = 39) occurring in two disjunct regions. These authors offered two evolutionary scenarios that could account for the four known karyotype groups in the *C. punctulatus* species complex. In the extreme version of the parallel scenario, all the karyotype variants arose independently from an ancestral population via centric fusions, and possibly more than once. In the alternate, sequential scenario, an ancestral karyotype (2n = 47) was the source of a chromosomal fusion (2n = 45), which in turn gave rise to an additional chromosomal fusion (2n = 43), and so on. Lo *et al.* [10] analysed the COII gene from members of the *C. punctulatus* species complex from 15 locations. Although their results upheld the serial reduction hypothesis, support for the relationships among the chromosomal lineages was not high. Taxa with the same chromosome number formed monophyletic groups, the exception being the two disjunct populations of the 2n = 39 karyotype.

**Figure 4:**
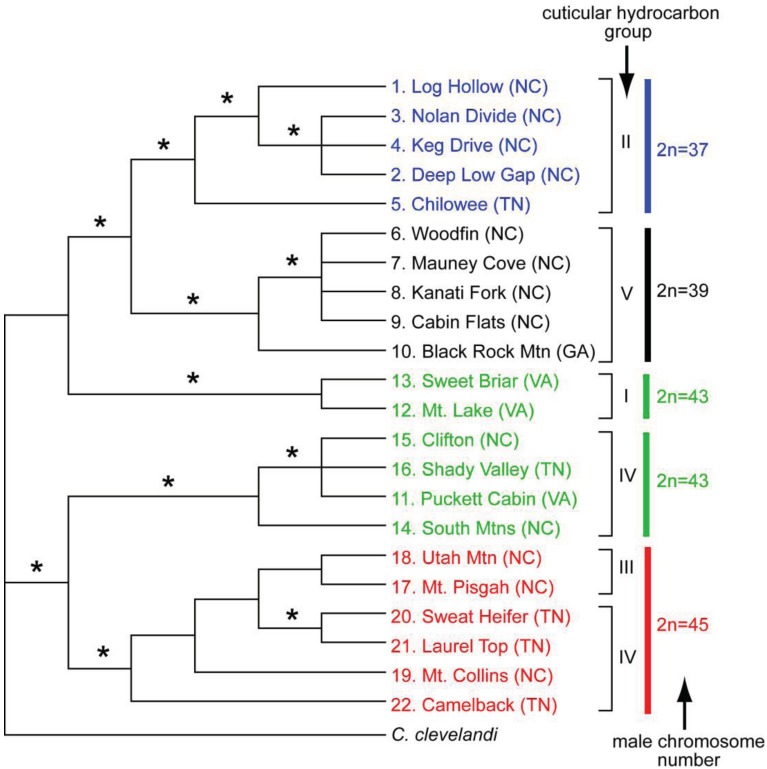
Strict consensus of three MP trees of the *Cryptocercus punctulatus* species complex from 22 locations derived from the combined datasets of mtDNA and nuclear DNA (re-drawn from the figure shown in Everaerts *et al.* [11]). Nodes with strong support (more than 90% Bayesian posterior probabilities and MP bootstrap probabilities) are indicated by an asterisk. Each terminal is labeled with the collection location number and name (shown in Figure 2c). Cuticular hydrocarbon group and the male chromosome number are also indicated.

Although molecular analyses of the karyotype groups in the eastern United States have resulted in three competing phylogenetic trees (43(39(45 + 37))) [37], (45(43(37 + 39))) [8,10], and (43(45(37 + 39))) [9], the emerging consensus is that the 2n = 37 and 39 groups are closely related and relatively apical. The 2n = 39 group appears to be divided into two geographically disjunct populations [39], and is likely not monophyletic [10]. Placement of the 2n = 43 and 45 groups within the tree varies among currently available analyses. Everaerts *et al.* [11] examined species limits by analyzing cuticular hydrocarbons, as well as mitochondrial and nuclear genes, in the four karyotype groups of *C. punctulatus* species complex. They found five distinct hydrocarbon phenotypes, but these were only partially congruent with chromosome number and therefore with purported species descriptions (Figure 4).

Molecular as well as cuticular hydrocarbon data indicate that *Cryptocercus* with a male 2n = 43 karyotype belong to at least two discrete, distantly related lineages. One is sister group to the 2n = 37 and 2n = 39 clade, and has a unique hydrocarbon profile. The other 2n = 43 lineage is sister group to the 2n = 45 samples, with cuticular hydrocarbons that group with four samples of the 2n = 45 lineage. Cuticular hydrocarbons of two other 2n = 45 samples diverge from this assemblage. Overall, the data suggest that cuticular hydrocarbons and chromosome number have some measure of evolutionary independence, so that neither is entirely reliable in delineating historical lineages. The Everaerts *et al.* [11] study is supportive of the parallel model of chromosomal evolution in the *C. punctulatus* species complex.

### 4.3. Biogeography of Nearctic Species

Based on the rate of evolution proposed above (see Section 1.2.), the endosymbionts of *C. clevelandi* and the eastern *C. punctulatus* species complex diverged around 45–58 MYA (Palaeocene-Eocene), and the divergence among endosymbionts of the eastern groups occurred at the beginning of the Miocene (18–24 MYA). These data are roughly congruent with the biogeographical discussion proposed by Nalepa *et al.* [7,20]. Here we revisit the biogeography of the Nearctic species, particularly the *C. punctulatus* species complex.

Nalepa *et al.* [20] pointed out that, although glaciers did not extend to the southeastern United States during the Quaternary, the area was nonetheless affected by the shifts in global atmospheric circulation patterns that caused glacial advances and retreats. Both landform and pollen records indicate that periglacial conditions prevailed in the Central and Southern Appalachian Mountains during the last full glacial interval (the Wisconsin Glacial Interval in North America), and were harsh in some locales [43,44]. The composition and location of vegetation changed, and boreal forests covered much of the region between 34° and 40° N latitude [44,45]. Like many detritivores, however, *Cryptocercus* likely would have had little problem weathering plant species shifts in Southern Appalachian forests; host choice is more dependent on decompositional status than on source tree species. *Cryptocercus* is also protected against climatic extremes, because an insulating buffer zone encapsulates the interior of large logs. These properties, among others, may explain the historical persistence of the genus [29,46].

Two factors may have been primary in influencing historic distributional shifts in Southern Appalachians *Cryptocercus* [20]. The first is absence of mature forest and thus coarse woody debris, and the second is adequate moisture. The former may have forced *Cryptocercus* from higher elevations during cold, wet, full glacial periods, and the latter may have restricted the cockroaches to mountaintops during the warmer, drier interglacials.

All mountains of the southeastern U.S. are below timber line today, but during glacial advances the tree-line in the Southern Appalachians was depressed to elevations ranging from 914 to 1500 m [47,48]. Mountain summits were covered by extensive areas of alpine tundra, with discontinuous regions of spruce and fir krumholtz [44,49]. Nalepa *et al.* [20] suggested that the lack of habitable logs at high elevations during this time would have forced *Cryptocercus* off mountains and into local refugia; the prevailing cool, moist climate concurrently made lower elevation forested sites more habitable for the cockroach. When glaciers began their undulating retreat, the warming climate allowed for the migration of species out of refugia, and forests could re-invade high altitude areas [44,49,50]. At low elevations, warmth, drought, and anthropogenic changes increasingly restricted mesic forest and *Cryptocercus* to favorable islands of habitat [44,51–54].

All four known karyotypes of *Cryptocercus* in the eastern United States are present in North Carolina between 35° 27′ and 35° 36′ N, where the Southern Appalachian Mountains are geographically widest and topographically the most complex [55]. Disconnected, differently oriented mountain ranges are separated by narrow, often riparian valleys frequently more than 900 m lower in elevation. The consequent variation in habitat types and potential for local habitat shifts during climatic changes assured many organisms of suitable but discontinuous refugia during glacial advance. *Cryptocercus* likely became spatially subdivided along with their habitat. If gene flow between refugial populations of the cockroach was suspended for a sufficient period of time, the insects would have differentiated *in situ*; consequently diversification was allopatric, because refuge formation is an ecologically vicariant process [56]. Nalepa *et al.* [20] noted that the winglessness of *Cryptocercus* would have accentuated population subdivision, because in organisms with restricted dispersal capability, ranges are known to be easily fragmented [57].

It is possible that the current parapatric boundaries between karyotypes of *Cryptocercus*, then, are relatively recent [20]. The most recent spread of Appalachian endemics out of refugia occurred during the past 20,000 years, the time interval since the last major glaciation [44]. However, the question of when the karyotypes actually differentiated is more problematic. Diversification of many species complexes has been linked to cycles of habitat expansion and contraction related to glacial advance and retreat. The first ice advance into the Appalachians equivalent to that of the late Wisconsin occurred in the Pliocene about 2.4 MYA; glaciation intensified in the middle and late Pleistocene [43]. Nonetheless, sequence divergence rates of the 16S rDNA of bacterial endosymbionts of the fat body suggest that divergence among the Southern Appalachian populations of *Cryptocercus* began at the onset of the Miocene (18–24 MYA) [20]. If so karyotype differentiations may have long predated the start of the ice ages [20].

## 5. Conclusions

Complex mountain topography in both Asia and North America strongly contributes to the known distribution of taxonomic groups of *Cryptocercus*. Glacial advances and retreats of the Tertiary and Quaternary influenced forests and consequently, the presence of the rotting log hosts required by genus. Anthropogenic land use changes have an impact on current distributions. The geographic origin of the genus is still unknown, and phylogenetic relationships among all described species need clarification. Asian species appear to be more morphologically distinct than those in Eastern North America, but the full range of species diversity on both continents requires further study. One approach to improve understanding of *Cryptocercus* spp. phylogeography may be to estimate divergence times among lineages using recently developed methods (e.g., Bayesian methods [58]).

## References

[1] Cleveland L.R., Hall S.R., Sanders E.P., Collier J. (1934). The wood-feeding roach *Cryptocercus*, its protozoa, and the symbiosis between protozoa and roach. Mem. Am. Acad. Arts Sci..

[2] Seelinger G., Seelinger U. (1983). On the social organization, alarm and fighting in the primitive cockroach *Cryptocercus punctulatus* Scudder. Zeitschrift für Tierpsychologie.

[3] Nalepa C.A. (1984). Colony composition, protozoan transfer and some life history characteristics of the woodroach *Cryptocercus punctulatus* Scudder (*Dictyoptera: Cryptocercidae*). Behav. Ecol. Sociobiol..

[4] Grandcolas P., Park Y.C., Choe J.C., Piulachs M.D., Belles X., D'Haese C., Farine J.P., Brossut R. (2001). What does *Cryptocercus kyebangensis*, n. sp. from South Korea about *Cryptocercus* evolution? A study in morphology, molecular phylogeny and chemistry of tergal glands (Dictyoptera, Blattaria, Polyphagidae). Proc. Acad. Nat. Sci. Phila..

[5] Park Y.C., Grandcolas P., Choe J.C. (2002). Colony composition, social behavior and some ecological characteristics of the Korean wood-feeding cockroach (*Cryptocercus kyebangensis*). Zool. Sci..

[6] Lo N., Eggleton P., Bignell D.E., Roisin Y., Lo N. (2011). Termite phylogenetics and co-cladogenesis with symbionts. Biology of Termites: A Modern Synthesis.

[7] Nalepa C.A., Byers G.W., Bandi C., Sironi M. (1997). Description of *Cryptocercus clevelandi* (*Dictyoptera: Cryptocercidae*) from the northwestern United States, molecular analysis of bacterial symbionts in its fat body, and notes on biology, distribution, and biogeography. Ann. Entomol. Soc. Am..

[8] Burnside C.A., Smith P.T., Kambhampati S. (2000). Three new species of the wood roach, *Cryptocercus* (*Blattodea: Cryptocercidae*) from the eastern United States. J. Kans. Entomol. Soc..

[9] Clark J.W., Hossain S., Burnside C., Kambhampati S. (2001). Coevolution between a cockroach and its bacterial endosymbiont: A biogeographical perspective. Proc. R. Soc. Lond. B..

[10] Lo N., Luykx P., Santoni R., Beninati T., Bandi C., Casiraghi M., Lu W.H., Zakharov E.V., Nalepa C.A. (2006). Molecular phylogeny of *Cryptocercus* wood-roaches based on mitochondrial COII and 16S sequences, and chromosome numbers in Palearctic representatives. Zool. Sci..

[11] Everaerts C., Maekawa K., Farine J.P., Shimada K., Luykx P., Brossut R., Nalepa C.A. (2008). The *Cryptocercus punctulatus* species complex (*Dictyoptera: Cryptocercidae*) in the eastern United States: Comparsion of cuticular hydrocarbons, chromosome number, and DNA sequences. Mol. Phylogenet. Evol..

[12] Park Y.C., Maekawa K., Matsumoto T., Santoni R., Choe J.C. (2004). Molecular phylogeny and biogeography of the Korean woodroaches *Cryptocercus* spp.. Mol. Phylogenet. Evol..

[13] Maekawa K., Park Y.C., Lo N. (2005). Phylogeny of endosymbiont bacteria harbored by the woodroach *Cryptocercus* spp. (*Cryptocercidae: Blattaria*): Molecular clock evidence for a late Cretaceous-early Tertiary split of Asian and American lineages. Mol. Phylogenet. Evol..

[14] Grandcolas P. (1999). Systematics, endosymbiosis and biogeography of *Cryptocercus clevelandi* and *C. punctulatus* (*Blattaria: Polyphagidae*) from North America: A phylogenetic perspective. Ann. Entomol. Soc. Am..

[15] Grandcolas P., Legendre F., Park Y.C., Belles X., Murienne J., Pellens R. (2005). The genus *Cryptocercus* in East Asia: Distribution and new species (*Insecta, Dictyoptedra, Blattaria, Polyphagidae*). Zoosystema.

[16] Grandcolas P. (2000). *Cryptocercus matilei* n. sp., du Sichuan de Chine (*Dictyoptera, Blattaria, Polyphaginae*). Rev. Française d'Entomol. (N.S.).

[17] Bey-Bienko G. (1938). On some new or interesting Asiatic *Blattodea*. Ann. Mag. Nat. Hist. 1.

[18] Nalepa C.A., Li L., Wen-Hua L., Lazell J. (2001). Rediscovery of the wood-eating cockroach *Cryptocercus primarius* (*Dictyoptera: Cryptocercidae*) in China, with notes on ecology and distribution. Acta Zootaxonomica Sin..

[19] Bey-Bienko G. (1935). Description of six new species of Palearctic Blattodea. Konowia.

[20] Nalepa C.A., Luykx P., Klass K.-D., Deitz L.L. (2002). Distribution of karyotypes of the *Cryptocercus punctulatus* species complex (*Dictyoptera: Cryptocercidae*) in the Southern Appalachians: Relation to habitat and history. Ann. Entomol. Soc. Am..

[21] Bandi C., Sironi M., Damiani G., Magrassi L., Nalepa C.A., Laudani U., Sacchi L. (1995). The establishment of intracellular symbiosis in an ancestor of cockroaches and termites. Proc. R. Soc. Lond. B.

[22] Grimaldi D. (1997). A fossil mantis (Insecta: Mantodea) in Cretaceous amber of New Jersey, with comments on the early history of the Dictyoptera. Am. Mus. Novit..

[23] Labandeira C.C. (1994). A compendium of fossil insect families. Milwaukee Public Mus. Contrib. Biol. Geol..

[24] Vrsansky P., Vishniakova V.N., Rasnitsyn A.P., Rasnitsyn A.P., Quicke D.L.J. (2002). Order Blattida Latreille, 1810. History of insects.

[25] Moran N.A., Munson M.A., Baumann P., Ishikawa H. (1993). A molecular clock in endosymbiotic bacteria is calibrated using the insect hosts. Proc. R. Soc. Lond. B.

[26] Raven P.H., Axelrod D.I. (1974). Angiosperm biogeography and past continental movements. Ann. Mo. Bot. Gard..

[27] Wolfe J.A. (1975). Some aspects of plant geography of the Northern Hemisphere during the late Cretaceous and Tertiary. Ann. Mo. Bot. Gard..

[28] Tiffney B.H. (1985). The Eocene North Atlantic Bridge: Its importance in Tertiary and modern phytogeography of the Northern Hemisphere. J. Arnold Arbor..

[29] Nalepa C.A., Bandi C. (1999). Phylogenetic status, distribution, and biogeography of *Cryptocercus*. Ann. Entomol. Soc. Am..

[30] Sher A. (1999). Traffic lights at the Beringian Crossroads. Nature.

[31] Asahina S. (1991). Notes on two small collections of the Blattaria from China and Korea. Akitu.

[32] Beckenbach A.T., Wei Y.W., Liu H. (1993). Relationships in the *Drosophila obscura* species group, inferred from mitochondrial cytochrome oxidase II sequences. Mol. Biol. Evol..

[33] Yi S.H., Yi S.S., Batten D.J., Yun H.S., Park S.J. (2003). Cretaceous and Cenozoic non-marine deposits of the Northern South Yellow Sea Basin, offshore western Korea: Palynostratigraphy and palaeoenvironments. Palaeogeogr. Palaeoclimatol. Palaeoecol..

[34] Park Y.A., Yi H.I. (1989). Late quaternary climatic changes and sea-level history along the Korean coasts. J. Coastal Res. Spec. Issue.

[35] Park Y.A. (1992). The changes of sea level and climate during the late Pleistocene and Holocene in the Yellow Sea egion. Korean J. Quat. Res..

[36] Park Y.A., Kim B.K., Zhao S. (1994). Sea level fluctuation in the Yellow Sea Basin. J. Korean Soc. Oceanogr..

[37] Atkinson T.H., Koehler P.G., Patterson R.S. (1991). Catalogue and Atlas of the Cockroaches (*Dictyoptera*) of North America North of Mexico. Misc. Publ. Entomol. Soc. Am..

[38] Kambhampati S., Luykx P., Nalepa C.A. (1996). Evidence for sibling species in *Cryptocercus punctulatus*, the wood roach, from variation in mitochondrial DNA and karyotype. Heredity.

[39] Brossut R., Nalepa C.A., Bonnard O., Le Quere J.L., Farine J.P. (1991). Tergal glands of male and female *Cryptocercus punctulatus* scudder (*Dictyoptera: Cryptocercidae*): Composition, sexual dimorphism, and geographic variation of secretion. J. Chem. Ecol..

[40] Cohen S.H., Roth L.M. (1970). Chromosome numbers of the Blattaria. Ann. Entomol. Soc. Am..

[41] Luykx P. (1983). XO-XX sex chromosomes and Robertsonian variation in the autosomes of the wood-roach *Cryptocercus punctulatus* (*Dictyoptera: Blattaria: Cryptocercidae*). Ann. Entomol. Soc. Am..

[42] White M.J.D. (1973). Animal Cytology and Evolution.

[43] Braun D.D. (1989). Glacial and periglacial erosion of the Appalachians. Geomorphology.

[44] Delcourt P.A., Delcourt H.R., Morse D.F., Morse P.A., Martin W.H., Boyce S.G., Echternacht A.C. (1993). History, evolution, and organization of vegetation and human culture. Biodiversity of the Southeastern United States: Lowland Terrestrial Communities.

[45] Watts W.A. (1970). The full-glacial vegetation of northwestern Georgia. Ecology.

[46] Nalepa C.A., Bignell D.E., Bandi C. (2001). Detritivory, coprophagy, and the evolution of digestive mutualisms in *Dictyoptera*. Insectes Soc..

[47] King P.B., Stupka A. (1950). The Great Smoky Mountains-their geology and natural history. Sci. Mon..

[48] Michalek D.D. (1969). Fanlike Features and Related Periglacial Phenomena of the Southern Blue Ridge. Ph.D. dissertation.

[49] Mills H.H., Delcourt P.A., Morrison R.B. (1991). Quarternary geology of the Appalachian highlands and interior low plateaus. Quarternary Nonglacial Geology.

[50] Webb T.I., Bartlein P.J. (1992). Global changes during the last 3 million years: Climatic controls and biotic responses. Annu. Rev. Ecol. Syst..

[51] Folkerts G.W. (2006). The range of the eastern woodeating cockroach *Cryptocercus punctulatus* Scudder (*Blattaria: Cryptocercidae*) in the United States. Entomol. News.

[52] Hardin J.W., Cooper A.W. (1967). Mountain disjuncts in the eastern Piedmont of North Carolina. J. Elisha Mitchell Sci. Soc..

[53] Martin P.S., Hubbs C.L. (1958). Pleistocene ecology and biogeography of North America. Zoogeography.

[54] Nalepa C.A. (2001). *Cryptocercus punctulatus* (*Dictyoptera: Cryptocercidae*) from monadnocks in the Piedmont of North Carolina. J. Entomol. Sci..

[55] White P.S., Buckner E.R., Pittillo J.D., Cogbill C.V., Martin W.H., Boyce S.G., Echternacht A.C. (1993). High-elevation forests: Spruce-fir forests, northern hardwoods forests, and associated communities. Biodiversity of the Southeastern United States.

[56] Patton J.L., da Silva M.N.F., Howard D.J., Berloucher S.H. (1998). Rivers, refuges and ridges. The geography of speciation of Amazonian mammalsl. Endless Forms. Species and Speciation.

[57] Vermeij G.J., Elliot D.K. (1986). Survival during biotic crises: The properties and evolutionary significance of refuges. Dynamics of Extinction.

[58] Drummond A.J., Ho S.Y.W., Phillips M.J., Rambaut A. (2006). Relaxed phylogenetics and dating with confidence. PLoS Biol..

